# Efficacy and safety data of subsequent entry biologics pertinent to nephrology practice: a systematic review

**DOI:** 10.1186/s40697-014-0034-5

**Published:** 2014-12-23

**Authors:** Judith Genevieve Marin, Marianna Leung, Clifford Lo, Nicole W Tsao, Daniel J Martinusen

**Affiliations:** St. Paul’s Hospital, Providence Healthcare, 1081 Burrard Street, Vancouver, British Columbia V6Z 1Y6 Canada; British Columbia Provincial Renal Agency, 1081 Burrard Street, Vancouver, British Columbia V6Z 1Y6 Canada; Faculty of Pharmaceutical Sciences, University of British Columbia, Vancouver, Canada; Royal Jubilee Hospital, Island Health Authority, 1952 Bay Street, Victoria, British Columbia V8R 1 J8 Canada

**Keywords:** Subsequent entry biologic, Rituximab, Darbepoetin, Tissue plasminogen activator, Epoetin alpha, Epoetin beta, Epoetin zeta, HX 575, Epoetin theta

## Abstract

**Background:**

Subsequent entry biologics (SEBs) may soon be a reality in Canadian nephrology practice. Understanding the worldwide experience with these agents will be valuable to Canadian clinicians.

**Objectives:**

To compare the efficacy and safety data between SEBs used in nephrology practice and their reference biologic.

**Design:**

Systematic review.

**Sources of information:**

Ovid MEDLINE, EMBASE, Cochrane Database of Systematic Reviews, Database of Abstracts of Review of Effects, Cochrane Central Register of Controlled Trials.

**Patients:**

Adult patients with chronic kidney disease (CKD).

**Methods:**

Our systematic review follows the process outlined by Cochrane Reviews. For efficacy data, all randomized controlled trials (RCTs), quasi-RCTs and observational trials in nephrology practice were included. For safety data, case series, case reports, review articles in nephrology practice and pharmacovigilance programs were included as well.

**Results:**

Only epoetin SEBs trials were published in the literature. Ten studies involving three different epoetin SEBs (epoetin zeta, HX575 and epoetin theta) were included. The mean epoetin dose used did not differ significantly between the SEBs and the reference product. For epoetin zeta and epoetin theta, the mean hemoglobin levels achieved in the studies were similar between the SEBs and the reference epoetin. The HX 575 studies reported a mean absolute change in hemoglobin within the predefined equivalence margin, when compared with the reference biologic. In terms of safety data, 2 cases of pure-red-cell aplasia were linked to the subcutaneous administration of HX 575. Otherwise, the rate of adverse drug reactions was similar when epoetin SEBs were compared with the reference biologic.

**Limitations:**

Our analysis is limited by the paucity of information available on SEB use in nephrology with the exception of epoetin SEBs. Methodological flaw was found in one of the epoetin zeta studies which accounted for 45% of pooled results.

**Conclusions:**

Little clinical difference was found between epoetin SEBs and the reference product. Although not deemed clinically important, the financial implication of a possible dose difference between epoetin zeta and reference product should be considered in pharmacoeconomic studies. Ongoing trials are expected to address the risk of pure-red-cell aplasia with HX 575.

## Abrégé

**Contexte:**

Il est possible que les produits biologiques ultérieurs (PBU) soient bientôt utilisés en néphrologie. Afin de guider la pratique en néphrologie au Canada, il est important de comprendre l’ensemble des expériences produites, à l’échelle mondiale, en matière d’efficacité et d’innocuité de ces agents.

**Objectifs:**

Comparer les données relatives à l’efficacité et l’innocuité entre les PBU utilisés en néphrologie et leurs médicaments biologiques de référence.

**Type d’étude:**

Revue systématique.

**Sources de données:**

La recherche en vue de la revue de littérature a été effectuée en interrogeant les bases de données suivantes : Ovid Medline, Embase, la base de données de revues systématiques Cochrane Reviews, la Database of Abstracts of Reviews of Effects, et la Cochrane Central Register Controlled Trials.

**Patients:**

Les patients d’âge adulte atteints de néphropathie chronique.

**Méthodes:**

Notre revue systématique suit la méthode suggérée par la Collaboration Cochrane (Cochrane Reviews). Pour les données se rapportant à l’efficacité, l’ensemble des essais randomisés contrôlés (ERC) et des modèles quasi expérimentaux et des études observationnelles du domaine de la néphrologie ont été comptabilisés. Pour les données se rapportant à l’innocuité, tous les ERC, les modèles quasi expérimentaux, les études observationnelles, les études ou séries de cas, ainsi que les revues d’articles en néphrologie clinique et de programmes en pharmacovigilance ont été comptabilisés.

**Résultats:**

Nous nous sommes attardés à l’époétine biologique ultérieure, puisqu’aucune documentation sur d’autres produits biologiques ultérieurs (PBU) n’était disponible. Nous avons utilisé dix études présentant trois époétines biologiques ultérieures différentes (époétine zeta, époétine HX575 et époétine thêta). Il n’existait pas de différence significative entre les doses moyennes des PBU et d’époétine biologique de référence. La dose moyenne d’époétine utilisée ne variait pas de façon significative entre les PBU et le produit de référence. Pour les époétines zeta et thêta, les taux moyens d’hémoglobine obtenus dans les diverses études entre les PBU et les époétines de référence étaient similaires. Les études se rapportant à l’époétine HX575 montraient un changement absolu du taux moyen d’hémoglobine à l’intérieur de l’intervalle d’équivalence prédéfini, lorsque comparé au médicament biologique de référence. En ce qui concerne les données d’innocuité, deux cas d’érythroblastopénie chronique acquise ont été liés à l’administration sous-cutanée de l’époétine HX575. Sinon, les taux d’effets indésirables recensés pour l’époétine biologique ultérieure et son médicament biologique de référence étaient similaires.

**Limites de l’étude:**

Notre analyse est limitée par la rareté de l’information accessible sur l’utilisation des PBU en néphrologie, à l’exception de l’époétine biologique ultérieure. Une faille sur le plan méthodologique a été retrouvée dans une des études sur l’époéine zeta. Celle-ci se rapportait à 45 % des résultats regroupés.

**Conclusions:**

Peu de différences sur le plan clinique ont été trouvées entre l’époétine biologique ultérieure et l’époétine de référence. Même si elles ne sont pas cliniquement significatives, les répercussions financières qui sont entraînées par la possible différence de dose entre l’époétine zeta et le produit de référence devraient être considérées dans les études pharmacoéconomiques. Il existe certaines préoccupations entourant les risques d’érythroblastopénie chronique acquise et l’époétine HX575, mais des essais présentement en cours tentent de faire le tour de la question.

**What was known before:**

SEBs pertinent to nephrology practice are available commercially in other countries. Epoetin SEBs have been compared with reference epoetins in clinical studies involving CKD patients.

**What this adds:**

This systematic review summarizes the efficacy data comparing epoetin SEBs to the reference epoetins in patients with CKD and provides an overview of the European experience in terms of safety data for epoetin SEBs. No other SEBs pertinent to nephrology practice have published data as yet.

## Why this report is important?

Subsequent entry biologics (SEBs) may soon be a reality in Canadian nephrology practice. A critical evaluation of the SEB trials will enable the Canadian nephrology community to make important decisions regarding the safe and effective use of these agents.

## Key messages

Little clinical difference was found between epoetin SEBs and the reference product; however, a non-clinically important dose difference between epoetin zeta and reference product should be noted. Pure-red-cell aplasia has been reported with the subcutaneous administration of HX 575.

## Implications for future research

Pharmacoeconomic studies should be conducted to assess the financial implication of a possible dose difference between epoetin zeta and reference product. Post-marketing surveillance is needed to provide a more precise estimate of pure-red-cell aplasia frequency and to establish the overall adverse reaction profile of all epoetin SEBs in clinical practice. Studies are needed for SEBs of other biologics commonly used in nephrology practice, such as darbepoetin, tissue plasminogen activator and rituximab.

## Introduction

Biologic medicines have contributed to the health of Canadians since the 1980s. In nephrology, erythropoiesis stimulating agents (ESAs), a biologic drug, have been the cornerstone of renal anemia treatment since epoetin alpha was marketed in the 1980s [1]. As patents expire for many of these products within this decade, subsequent entry biologics (SEBs), or the “generic” of the innovator biologic, will be entering the Canadian market. For example, the Canadian patent for epoetin alpha expired in May 2014 and epoietin SEBs are expected to enter the Canadian market within the next year. They bring the opportunity to reduce health care costs, but pose unique challenges. Even if SEBs are highly similar to the innovator product, the small differences have the potential to translate into clinical differences in efficacy, safety and immunogenicity [2]. To improve our understanding around these new drugs, which might be available on the Canadian market in a near future, we conducted a systematic review with the following objectives:

1. To compare the efficacy data between SEBs used in nephrology practice and their reference biologic
2. To compare the safety data between SEBs used in nephrology practice and their reference biologic with regards to expected side effects (common or rare)
3. To summarize any unexpected side effects reported in the literature and pharmacovigilance programs for SEBs used in nephrology practice

## Methods

This systematic review follows the process outlined by Cochrane Reviews.

### Types of studies

For efficacy data, all randomised controlled trials (RCTs), quasi-RCTs and observational trials in nephrology practice were included. For safety data, all randomised controlled trials (RCTs), quasi-RCTs, observational trials, case series, case reports, review articles in nephrology practice and pharmacovigilance programs were included.

### Types of participants

For efficacy and safety data, adult and pediatric patients with CKD were included.

### Types of interventions

All trials evaluating the use of any SEBs were included, whether the intervention was tested on its own or head-to-head with the reference biologic. Specifically, a comprehensive literature search was conducted for SEBs of the following reference biologics:

1. epoetin
2. darbepoetin
3. rituximab
4. tissue plasminogen activator (tPA)

### Search methods for identification of studies

Relevant articles were obtained from Ovid MEDLINE <1946-2013 December 06 > and EMBASE <1974 to 2013 December 06 > electronic sources using the search terms biosimilar pharmaceuticals, subsequent entry biologics or follow on biologics for the drugs of interest: epoetin, darbepoetin, rituximab, and tissue plasminogen activator. In addition, the following databases were searched using the same search terms: Cochrane Database of Systematic Reviews (2005 to December 2013); Database of Abstracts of Review of Effects (December 2013); Cochrane Central Register of Controlled Trials (December 2013). The reference lists of review articles and relevant trials were also used to identify additional clinical trials. There was no language restriction.

### Data collection and analysis

The search strategies described above were used to obtain titles and abstracts of studies that might be relevant to this review. The titles and abstracts, and full text when necessary, were screened independently by J.G.M. and M.L., who excluded studies that were not applicable based on the above inclusion criteria; however, studies and reviews that potentially included relevant data or information on trials were included for full-text screening. Data extraction was carried out by the same reviewers using standardized Cochrane data extraction format. It was planned that studies reported in non-English language journals (if any) would be translated before assessment. Where more than one publication of one trial existed, only the publication with the most complete data was included. Disagreements were resolved by consensus between the two reviewers.

### Study quality

The quality of included studies was assessed independently by the same two reviewers, without blinding to authorship or journal, using the checklist developed by the Cochrane Group [3]. Discrepancies were resolved by consensus. The quality items assessed included allocation concealment, blinding of participants, investigators and outcome assessors, intention-to-treat analysis, and the completeness of follow-up.

### Statistical assessment

Results were expressed as a risk ratio (RR) with 95% confidence intervals (CI) for all categorical outcomes of the individual studies. Data were pooled using random effects and fixed effects models. Where continuous scales of measurement were used to assess the effects of treatment (epoetin dose used, hemoglobin values), the mean difference (MD) was used. Heterogeneity was analysed using a chi-squared test on N-1 degrees of freedom, with an alpha of 0.05 used for statistical significance and the I^2^ statistic. I^2^ values of 25%, 50% and 75% represent low, medium and high levels of heterogeneity. Subgroup analyses were planned to explore how possible sources of heterogeneity (hemodialysis versus non-dialysis CKD population, intravenous versus subcutaneous administration) might influence treatment effect. It was also planned that if sufficient RCTs were identified, an attempt would be made to assess funnel plot asymmetry due to small study effect, as this may be indicative of publication bias. Results are presented for each individual SEB.

## Results

No published clinical trials involving darbepoietin, rituximab or tPA were identified. As of December 2013, 18 human clinical trials involving SEB rituximab are registered with European Medicines Agency Clinical Trial Register (EUDRACT) or with the National Institute of Health at Clinicaltrials.gov [4,5]. Of these, 11 trials are conducted in patients with rheumatoid arthritis and seven in patients with hematological malignancies. Nine are already in phase III comparing the rituximab SEBs to the innovator product with four being open label extension trials evaluating efficacy and/or safety parameters. None of these trials has published preliminary results. Two of the rheumatoid arthritis trials (TL011 by TEVA and SAIT101 Samsung Biologics) and two oncology trials (MK8808 by Merck & Co. and CT-P10 by Celltrion) were halted prematurely, while two Sandoz trials involving GP-2013 have not had any updates on the Clinicaltrial.gov website for over two years. Exact reasons for early termination of the mentioned studies have not been given.

**Figure 1:**
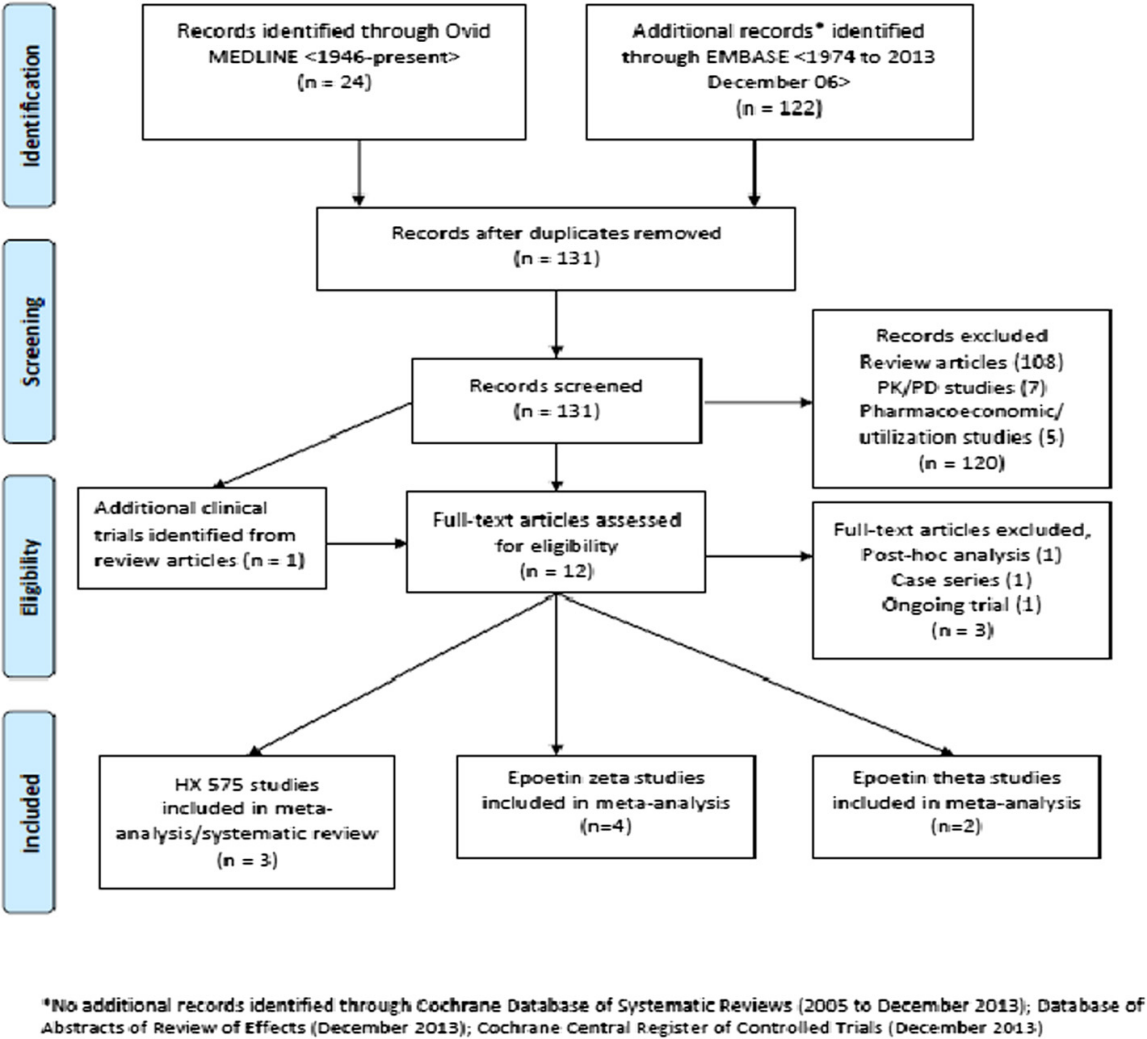
**Results of a literature review performed to identify clinical trials involving epoetin SEBs.**

Currently, two biosimilar molecules of epoetin alpha are available on the European market: epoetin zeta and HX575 [6]. Although developed as a stand-alone product, epoetin theta is clinically considered to be a biosimilar by many authors [7]. Table 1 provides the chemical denominations and brand names of the biosimilar epoetins marketed in EU. Since this review summarizes clinical outcomes related to biosimilar ESAs, results on epoetin theta have been incorporated into our report. The literature search retrieved 131 reports of SEB epoietin, of which 120 were excluded. Analysis of the 11 remaining articles identified 12 studies published in 12 articles which were analysed in full-text: four studies (2147 patients) were on epoetin zeta, three studies (2510 patients) were on HX575, and two studies (557 patients) were on epoetin theta. The search results are summarized in Figure 1. Reasons for exclusion of studies included non-CKD topics, duplicate reports, case reports, abstracts or methodology used that precluded analysis. A summary table of the included studies is presented at Table 2.

**Table 1:** **Summary of biosimilar epoetins marketed in the European Union**

**Chemical denominations**	**INN**	**Brand names**
**HX575**	Epoetin	Binocrit®
		Abseamed®
		Epoetin Alfa Hexal®
**SB309**	Epoetin zeta	Silapo®
		Retacrit®
**XM01 (not licensed as a biosimilar in EU)**	Epoetin theta	Eporatio®
		Biopoin®
		Ratioepo®

**Table 2:** **Summary of the studies included in the systematic review**

**Authors (year)**	**Design**	**Population**	**Enrolment**	**Intervention**	**Follow-up**	**Outcomes**
**HX 575**						
Haag-Weber et al. 2009 [15]	R DB multicentre parallel group equivalence study	HD patients	I: 314 C: 164	HX 575 vs. Epoetin alpha IV at a 1:1 dose conversion	56 weeks	1: Occurrence of anti-Epoetin Abs and the evaluations of ADR
Haag-Weber et al. 2012 [16]	RCT DB	CKD Stage 3-4	I: 175 C: 163	HX 575 SC 75 IU/kg/week vs. Epoetin alpha SC 75 IU/kg/week	26 weeks	1: Occurrence of anti-Epoetin Abs and the evaluations of ADRs
						Stopped early due to safety issue
Horl et al. 2012 [17]	Multicentre prospective single arm study	CKD patients on dialysis or not	I: 745	HX 575 IV 3 times/week	26 weeks	1: Occurrence of anti-Epoetin Abs and the evaluations of ADRs
**Epoetin zeta**						
Baldamus et al. 2008 [12]	Multicentre non-controlled, follow-up	HD patients	I:745	Epoetin zeta IV 1–3 times/week	56 weeks (108 weeks for Bulgarian subgroup)	1: Occurrence of anti-Epoetin Abs and the evaluations of ADRs
Krivoshiev et al. 2008 [8]	RCT DB multicentre	HD patients	I:305 C:304	Epoetin zeta IV 1–3 times/week vs. Epoetin alpha IV 1–3 times/week	24 weeks	1: Dose of epoetin/kg /week; Hb during the last 4 weeks of treatment; proportion of patients with treatment success, increase in Hb over time, proportion of patients with maintenance success, Hb during each 4-week interval, proportion of patients with an increase in Hb of > 1 g/dL for 4 weeks, percentage of Hb > 10 g/dL, percentage of HCT measurements > 30%, proportion of patients needing blood transfusion
Krivoshiev et al. 2010 [9]	RCT DB multicentre	HD patients	I: 232 C: 230	Epoetin zeta SC vs. Epoetin alpha SC	28 weeks	1: Dose of epoetin/kg/week; Hb during the last 4 weeks of treatment; mean HCT; proportion of patients with any permanent or transient changes in Hb > 1 g/dL; proportion of patients with any permanent or transient dose change; proportion of patients with any Hb outside the target range; incidence of blood transfusion
Lonneman et al. 2011 [10]	Observational single centre	HD patients	I: 18	Epoetin zeta IV	26 weeks	1: Dose of epoetin/kg/week ;Hb during the last 4 weeks of treatment; incidence of Hb > 13 g/dL, the ratings of local and general tolerability, the occurrence of anti-EPO Abs and evaluation of ADRs
Wizemann et al. 2008 [11]	DB cross-over study multicentre	HD patients	I: 155 C:158	Epoetin zeta IV 1–3 times/week and Epoetin alpha IV 1–3 times/week	12 weeks x 2	1: Dose of epoetin/kg/week; Hb during the last 4 weeks of treatment; HCT levels; proportion of patients with any permanent or transient changes in Hb > 1 g/dL; proportion of patients with any permanent or transient dose change; proportion of patients with any Hb measurement outside the target range; incidence of blood transfusion
**Epoetin theta**						
Gertz et al. 2010 [18]	Multicentre DB parallel-group non-inferiority controlled trial	HD patients	I:180 C: 90	Epoetin theta IV vs. Epoetin beta IV; 1:1 dosage conversion	26 weeks	1: Change in Hb from baseline to end of treatment
Gertz et al. 2012 [19]	Multicentre DB parallel-group non-inferiority controlled trial	CKD Stage 3–5 non on dialysis	I: 193 C: 95	Epoetin theta SC 1 time/week vs. Epoetin beta SC 1 time/week	26 weeks	1: Change in Hb from baseline to end of treatment

R = randomized; DB = double-blind; HD = hemodialysis; I = Intervention; C = Control; IV = intravenous; SC = subcutaneous; 1: Study primary outcome; Abs = Antibodies; ADRs = Adverse drug reactions; RCT = Randomized controlled trial; CKD = Chronic kidney disease; Hb = haemoglobin; HCT = Hematocrit.

1. Characteristics of studies
  Five clinical trials involving epoetin zeta were identified: two RCTs [8,9], one observational [10], one cross-over [11], and one long-term follow-up [12]. Two studies were excluded: a case report and a post-hoc analysis [13,14]. Of the comparison trials, three compared epoetin zeta to epoetin alpha [8,9,11] and one compared epoetin zeta to other ESAs after a switch-over [10]. All but one trial evaluated intravenous administration [8,10-12]; Krivoshiev 2010 evaluated subcutaneous route of administration [9]. All studies were conducted in hemodialysis patients.
  Three clinical trials involving epoetin HX575 were identified: two RCTs [15,16] and one prospective single-arm study [17]. All the included trials compared epoetin HX575 to epoetin alpha. Two out of the three studies evaluated intravenous administration of HX575 [16,17]. One study involving subcutaneous administration of HX575 was conducted in stage III to IV CKD patients [15].
  Two RCTs involving epoetin theta were identified [18,19]. No studies were excluded. Both studies compared epoetin theta to epoetin beta. One trial evaluated intravenous administration in hemodialysis patients [19] and the other trial evaluated the subcutaneous route in patients with CKD not yet receiving dialysis [18].
2. Risk of bias in included studies
  Of the five epoetin zeta studies, allocation concealment was adequate in two (40%) studies [8,11], only three (60%) studies blinded participants and investigators [8,9,11], and per protocol analyses were used for efficacy analysis in the epoetin zeta RCTs [8,9,11]. The percentage of patients lost to follow-up ranged from 0% to 9.9% [8-12].
  For the HX 575 studies, allocation concealment was unclear and participants and investigators were blinded in two out of three studies [15,16]; the third study was an unblinded prospective single-arm study [17]. An intention to treat (ITT) analysis was used in one study [17] while the other two studies used ITT analysis for safety data [15,16]. The percentage of patients lost to follow-up was reported in two studies to be 15.7% and 17.8% [15,16]. The percentage of patients lost to follow-up was not reported in the INJ-17 study since the study was stopped early [16].
  For the two epoetin theta studies, allocation concealment and blinding of participants and investigators were adequate in both (100%) studies, and an ITT analysis was used for safety analysis and per protocol analysis was used for efficacy data [18,19]. The number of patients lost to follow-up was 0/288 and 1/270. Of note, the EMA guidelines recommend the per protocol analysis as the primary analysis in equivalence/non-inferiority trials. ITT analyses are considered as sensitivity analysis and were performed for all clinical trials.
3. Effects of interventions
  There were no significant differences in the results of analyses performed using random and fixed effects models. The results presented below refer to those obtained using a random effects model. Subgroup analyses were not performed as the small number of patients and studies made the power of these analyses too small.

i. Mean epoetin dose used

**Figure 2:**
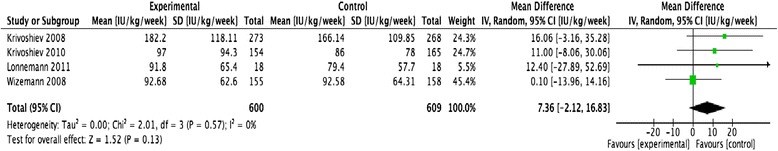
**Mean epoetin dose used in studies comparing epoetin zeta and reference.**
  Of note, three of the four studies showed that a higher dose was required with epoetin zeta compared with epoetin alpha to maintain similar hemoglobin levels [8-10], although this finding was not statistically significant in any of the studies. The remaining study was a crossover study by Wizemann et al. that accounted for 45% of the weighted result [11]. The outcomes from this study were measured during the treatment phase immediately after the switch-over and the concerns with carry-over effects from the first to the second treatment phase cannot be discounted. The application to the EMA provided additional analyses, including a comparison of hemoglobin values and epoetin doses over the last 4 weeks of each treatment period, as an attempt to minimise carry over effects from previous epoetin treatment. The equivalence margins were met with this new post-hoc analysis according to the EMA Scientific Report, but no details were given in the report that could be included in our meta-analysis [20]. When we excluded the Wizemann study because of the cross-over design, the pooled mean dose was 13 IU/kg/week (95% CI 0.57, 26); p = 0.04 (Figure 3) higher in the epoetin zeta arm.

1. Epoetin zeta
  No statistically significant differences were found between the mean epoetin dose. The pooled mean dose difference was 7.36 IU/kg/week (95% CI −2.12, 16.83); p = 0.13 (Figure 2).

**Figure 3:**
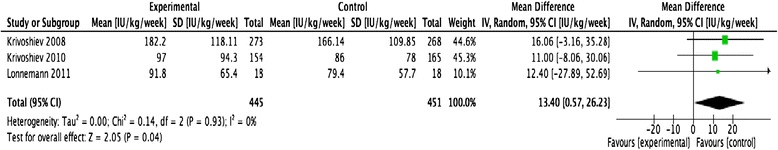
**Mean epoetin dose used in studies comparing epoetin zeta and reference epoetins excluding the study by Wizemann et al.**
2. HX 575
  No statistically significant differences were found between the mean epoetin doses used, which was 3.3 IU/kg/week (95% CI −4.0, 11); p = 0.38 (Figure 4).

**Figure 4:**
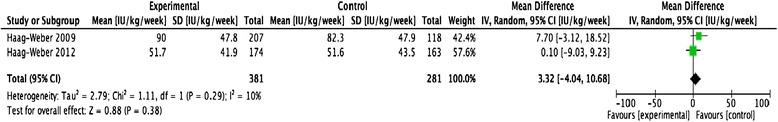
**Mean epoetin dose used in studies comparing HX575 and reference epoetin alpha.**
3. Epoetin theta
  No statistically significant differences were found between the mean epoetin doses used, which was −1.3 IU/kg/week (95% CI −8.7, 6.1); p = 0.73 (Figure 5).

**Figure 5:**
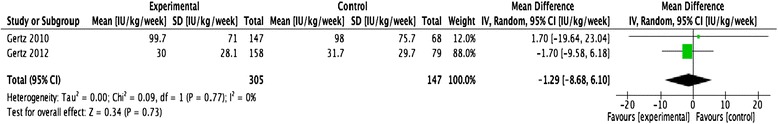
**Mean epoetin dose used in studies comparing epoetin theta and reference epoetin beta.**

ii. Mean hemoglobin levels

1. Epoetin zeta
  A statistically significant difference in mean hemoglobin level was achieved, with the control group having a higher mean haemoglobin level than the epoetin zeta group by 0.12 g/dL (or 1.2 g/L) (p = 0.03) (Figure 6). However, the absolute difference is small, and is unlikely of clinical importance.

**Figure 6:**
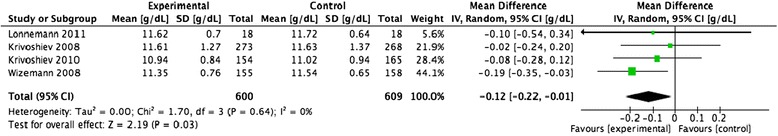
**Mean hemoglobin levels achieved in studies comparing epoetin zeta and reference epoetins.** Note: unit g/L = 10 x g/dL.
2. HX575
  None of the HX 575 studies used mean hemoglobin level as a study endpoint. However, , the mean hemoglobin levels for the Haag-Weber et al. 2009 study were included in the EMA application and varied between 11.6 to 11.9 g/dL (or 116 to 119 g/L) for the HX575 group and 11.7 to 12.1 g/dL (or 117 to 121 g/L) for the reference group over the course of the study [21]. The authors of the 3 studies were contacted but did not provide further information; therefore, a pooled analysis could not be performed.
3. Epoetin theta
  Mean difference in hemoglobin level was −0.01 g/dL (95% CI −0.18, 0.17) or −0.1 g/L (95% CI −1.8, 1.7), p = 0.95 (Figure 7).

**Figure 7:**
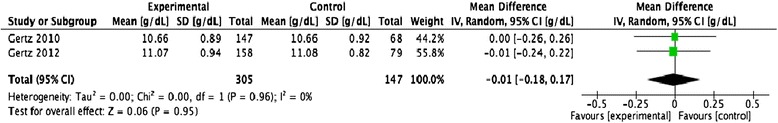
**Mean hemoglobin levels achieved in studies comparing epoetin theta and reference epoetin beta.** Note: unit g/L = 10 x g/dL.

iii. Mean absolute change in hemoglobin

1. HX575

**Figure 8:**
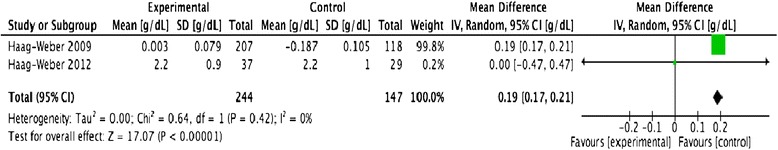
**Mean absolute change in hemoglobin levels in studies comparing HX575 and reference epoetin alpha.** Note: unit g/L = 10 x g/dL.

The mean absolute change in hemoglobin levels between the screening period and the evaluation period was reported in two HX 575 studies. An equivalence margin of ± 0.5 g/dl (or ± 5 g/L) in hemoglobin was chosen for the demonstration of comparable efficacy. As shown in Figure 8, the CI is within the predefined equivalence margin.

4. Safety data

I. Clinical trials

1. Epoetin zeta
  The presence of anti-erythropoietin antibodies was found at the screening phase but no cases of PRCA were reported [8,11,12]. In addition, no study found a difference in mortality between groups [8,9,11]. With regards to toxicity, similar rates of adverse effects were reported compared with the reference biologic [8-12].
2. HX575
  Subcutaneous administration of HX575 was linked to two cases of PRCA [16]; as such, HX575 is not approved for subcutaneous administration in people with chronic renal failure in Europe [21]. In addition, no study found a difference in mortality between the groups [15-17]. With regards to toxicity, similar rates of adverse effects were reported compared with the reference biologic, e.g., headache, hypertension, thrombosis [15-17].
3. Epoetin theta
  No cases of anti-erythropoietin antibodies or PRCA were reported [18,19] nor was there a difference in mortality rates [18,19]. With regards to toxicity, similar rates of adverse effects were reported compared to the reference biologic (headache, hypertension, thrombosis [18,19].

II. European pharmacovigilance data

EudraVigilance is a system designed to collect reports of suspected side effects and is used to evaluate the benefits and risks of a medication during its development and for monitoring its safety following market authorisation in the European Economic Area (EEA) [22]. Pharmaceutical companies that hold the marketing authorisation of a medication as well as national medicines regulatory authorities are legally required to submit reports of suspected side effects that occurred in the EEA to EudraVigilance. This includes reports received from healthcare professionals and patients. The pharmaceutical companies are also required to provide information on all serious unexpected adverse drug reactions that occurred in non-EEA countries where they hold a marketing authorization.

Table 3 summarizes all the immunological and cardiovascular serious adverse drug reactions reported in EudraVigilance as of January 1^st^ 2014 for the biosimilar epoetins currently on the EU market. Note that each case reported may contain one or multiple suspected ADRs and that the total number of cases represents the total number of reports submitted to EudraVigilance, for a specific marketed biosimilar.

**Table 3:** **Summary of the serious ADRs reported in EudraVigilance database (as per January 2014)** [22]

**Active ingredient**	**Brand name**	**Serious ADR reports**
HX 575	Abseamed®	**10 cases total**
		1 case of PRCA
		1 case of anemia
		2 cases of decreased hemoglobin
		2 cases of convulsion/epilepsy
		1 case of DVT
	Binocrit®	**36 cases in total**
		1 case of anemia
		2 cases of PRCA
		4 cases of decreased hemoglobin
		1 case of retinal artery occlusion
		3 cases of chest pain
		2 cases of pulmonary embolism
		2 cases of hypertension
		1 case of angiopathy
	Epoetin Hexal®	**4 cases in total**
		3 cases of decreased hemoglobin
		1 case of PRCA
		1 case of heart failure
Epoetin zeta	Retacrit®	**39 cases in total**
		1 case of anemia
		2 cases of decreased hemoglobin
		2 cases of myocardial infarction
		4 cases of death
		2 cases of drug ineffectiveness
		1 case of stroke
		1 case of convulsion
		1 case of carotid artery stenosis
		1 case of pulmonary embolism
		1 case of hypertensive crisis
		2 cases of thrombosis
	Silapo®	**6 cases in total**
		1 case of PRCA
		1 case of deceased hemoglobin
		2 cases of drug ineffectiveness
		1 case of hypertension crisis
Epoetin theta	Eporatio®	**7 cases in total**
		3 cases of PRCA
		1 case of anemia
		1 case of decreased hemoglobin
		1 case of angina
		1 case of epilepsy
		3 cases of drug ineffectiveness
	Biopoin®	**1 case in total**
		1 case of PRCA

ADRs = Adverse drug reactions; PRCA = Pure red cell aplasia.

N.B.: Each reported case may contain one or multiple suspected ADRs. Only ADRs commonly attributed to epoetin therapy are listed above.

## Discussion

This literature review found no clinically important differences in efficacy between the currently available SEB epoetins (epoetin zeta, HX575 and epoetin theta) and the reference ESA (epoetin alpha, epoetin beta and darbepoetin). However, some limitations related to the studies involving epoetin zeta should be noted. The Wizemann study [11], which accounts for 45.4% of the weighted pooled result, did not allow enough time for dose titration to achieve steady state in a crossover design. In addition, the investigators claimed that there was higher protein content due to overfilling (9% over the labelled amount of protein) noted with epoetin alpha compared with about 1% with epoetin zeta [20]. However, all syringes remained within the 80 to 125% of the stated dose allowed with the European Pharmacopoeia monograph for erythropoietin. The applicant used a correction factor and provided additional analyses to the EMA to adjust epoetin dosage for inter-batch variability in bioactivity and protein content. With the introduction of a correction factor, the 95% CI fell (−24, 17 IU/kg/week) in the Krivoshiev 2008 study, but the CI widened (3.1-14 IU/kg/week) in the Wizemann study [20]. The difference in syringe content may be explained by the use of different bioassays for determination of bioactivity for the test product (normocythaemic mouse bioassay used in the EU) and the reference product (exhypoxic polycythaemic mouse bioassay used in the USA). Lastly, the acceptable equivalence margin was modified post-hoc from ± 14 IU/kg/week to ± 45 IU/kg/week by the applicant to EMA due to a misreading of the European Public Assessment Report (EPAR) that listed the no-effect dosage of Dynepo® as 15 IU/kg given 3 times weekly but was misread by the study investigators as once weekly. This newly proposed acceptance range was supported by the literature, accepted by the EMA and deemed clinically unimportant. The upper limit of the CI for the dose difference is 17 IU/kg/week if the Wizemann study was included compared with 26 IU/kg/week if the Wizemann study was not included. The financial implication of a possible dose difference can be large when summed over populations and over years and should be considered by those who negotiate contracts for payers. The financial impact analysis is illustrated in the Tornado diagram in the paper by Tsao N et al. [23].

As for HX575, the mean absolute change in hemoglobin was statistically significant in favour of the control group [15]. However, it is within the pre-defined equivalence margin. Another study in healthy volunteers randomized to receive HX575 or Epogen® also pointed to this possible difference in efficacy [24]. This study determined that the two products were bioequivalent, even though HX575 exposure was approximately 10% lower [24]. This difference is difficult to ascertain since the information is quite sparse. The ongoing trials with HX575 should help elucidate this issue.

In terms of toxicity, this review found no differences between biosimilar epoetins (epoetin zeta, HX575 and epoetin theta) and the reference epoetin with the exception of two cases of PRCA, one confirmed and one possible, reported with the subcutaneous administration of HX575 [16]. These cases were attributed to the increased concentration of tungsten in the prefilled syringes causing denaturation of epoetin molecules and subsequent formation of immunogenic aggregates [16]. Subcutaneous administration is currently not an approved route of administration for HX575 in Europe [21] but there are ongoing trials to further evaluate the safety of subcutaneous administration of HX575. Since its launch in March 2012, patient exposure to HX575 has been estimated to be 134,928 patients-years, without any indication of increased immunogenicity [1,25]. Subcutaneous administration for both epoetin zeta (n = 232) and epoetin theta (n = 193) have been studied and no increased risk of PRCA have been found.

## Conclusion

Based on currently-available published information, epoetin zeta and epoetin theta appear to have similar efficacy to that of the reference ESA and no evidence of an increased risk of PRCA or adverse reactions. Although not considered clinically important or statistically significant in the clinical trials, the higher dose requirement for epoetin zeta to achieve target hemoglobin should be noted. Further studies should be conducted to confirm the dose difference with epoetin zeta, and pharmacoeconomic studies should be conducted to examine financial implications. The current available evidence is inadequate and further studies are required to clarify the risk of subcutaneous administration of HX575. Furthermore, post-marketing surveillance is needed to provide a more precise estimate of PRCA frequency and to establish the overall adverse reaction profile of all epoetin SEBs in clinical practice.
